# Medicine procurement and the use of flexibilities in the Agreement on Trade-Related Aspects of Intellectual Property Rights, 2001–2016

**DOI:** 10.2471/BLT.17.199364

**Published:** 2018-02-05

**Authors:** Ellen FM ‘t Hoen, Jacquelyn Veraldi, Brigit Toebes, Hans V Hogerzeil

**Affiliations:** a Global Health Unit, Department of Health Sciences, University Medical Centre Groningen, University of Groningen, PO Box 30.001, Groningen, 9700 RB, the Netherlands.; b Faculty of Law, University of Groningen, Groningen, the Netherlands.; c Department of International Law, University of Groningen, Groningen, the Netherlands.

## Abstract

Millions of people, particularly in low- and middle-income countries, lack access to effective pharmaceuticals, often because they are unaffordable. The 2001 Ministerial Conference of the World Trade Organization (WTO) adopted the Doha Declaration on the TRIPS (Trade-Related Aspects of Intellectual Property Rights) Agreement and Public Health. The declaration recognized the implications of intellectual property rights for both new medicine development and the price of medicines. The declaration outlined measures, known as TRIPS flexibilities, that WTO Members can take to ensure access to medicines for all. These measures include compulsory licensing of medicines patents and the least-developed countries pharmaceutical transition measure. The aim of this study was to document the use of TRIPS flexibilities to access lower-priced generic medicines between 2001 and 2016. Overall, 176 instances of the possible use of TRIPS flexibilities by 89 countries were identified: 100 (56.8%) involved compulsory licences or public noncommercial use licences and 40 (22.7%) involved the least-developed countries pharmaceutical transition measure. The remainder were: 1 case of parallel importation; 3 research exceptions; and 32 non-patent-related measures. Of the 176 instances, 152 (86.4%) were implemented. They covered products for treating 14 different diseases. However, 137 (77.8%) concerned medicines for human immunodeficiency virus infection and acquired immune deficiency syndrome or related diseases. The use of TRIPS flexibilities was found to be more frequent than is commonly assumed. Given the problems faced by countries today in procuring high-priced, patented medicines, the practical, legal pathway provided by TRIPS flexibilities for accessing lower-cost generic equivalents is increasingly important.

## Résumé

Des millions de personnes, en particulier dans les pays à revenu faible et intermédiaire, ne peuvent accéder à des produits pharmaceutiques efficaces, souvent en raison de leur prix trop élevé. La Conférence ministérielle de 2001 de l'Organisation mondiale du commerce (OMC) a adopté la Déclaration de Doha sur l'Accord sur les ADPIC (aspects des droits de propriété intellectuelle qui touchent au commerce) et la santé publique. Cette déclaration a reconnu les implications des droits de propriété intellectuelle, aussi bien pour le développement de nouveaux médicaments que pour le prix des médicaments. Elle a détaillé des mesures, appelées flexibilités des ADPIC, que peuvent prendre les Membres de l'OMC pour assurer l'accès de tous aux médicaments, comme l'octroi de licences obligatoires aux brevets de médicaments et la mesure de transition pharmaceutique des pays les moins avancés. Le but de cette étude était d'examiner le recours aux flexibilités des ADPIC pour accéder à des médicaments génériques moins coûteux entre 2001 et 2016. Dans l'ensemble, 176 cas de recours possible aux flexibilités des ADPIC par 89 pays ont été relevés: 100 (56,8%) concernaient des licences obligatoires ou des licences d'utilisation publique à des fins non commerciales et 40 (22,7%) concernaient la mesure de transition pharmaceutique des pays les moins avancés. Quant aux autres, il s'agissait d'un cas d'importation parallèle, de 3 exceptions de recherche et de 32 mesures sans lien avec des brevets. Sur ces 176 cas, 152 (86,4%) ont été mis en œuvre. Ils portaient sur des produits destinés à traiter 14 maladies différentes. Cependant, 137 (77,8%) concernaient des médicaments contre le virus de l'immunodéficience humaine et le syndrome d'immunodéficience acquise ou des maladies apparentées. Le recours aux flexibilités des ADPIC s'est révélé plus fréquent que ce que l'on supposait. Étant donné les problèmes que rencontrent actuellement certains pays pour se procurer des médicaments brevetés à prix élevé, le cadre pratique et juridique offert par les flexibilités des ADPIC pour accéder à des équivalents génériques moins coûteux revêt une importance de plus en plus capitale.

## Resumen

Millones de personas, particularmente en países de ingresos bajos y medios, carecen de acceso a medicamentos efectivos, habitualmente porque no pueden pagarlos. La Conferencia Ministerial de 2001 de la Organización Mundial del Comercio (OMC) adoptó la Declaración de Doha relativa al Acuerdo sobre los ADPIC (Aspectos de los Derechos de Propiedad Intelectual relacionados con el Comercio) y la Salud Pública. La declaración reconoció las implicaciones de los derechos de propiedad intelectual para el desarrollo de nuevos medicamentos y el precio de los mismos. La declaración describió medidas, conocidas como flexibilidades de los ADPIC, que los Miembros de la OMC pueden tomar con el objetivo de asegurar el acceso a los medicamentos para todos. Estas medidas incluyen concesión obligatoria de licencias de patentes de medicamentos y la medida de transición farmacéutica de países menos desarrollados. El objetivo de este estudio fue documentar el uso de las flexibilidades de los ADPIC para acceder a medicamentos genéricos de precio inferior entre el 2001 y el 2016. En general, se identificaron 176 casos de posibles usos de las flexibilidades de los ADPIC: 100 (56.8%) implicaron licencias obligatorias o licencias de uso público no comercial y 40 (22.7%) apelaron a la medida de transición farmacéutica de países menos desarrollados. El resto fue: 1 caso de importación paralela; 3 excepciones de investigación; y 32 medidas no relacionadas con patentes. De los 176 casos, 152 (86.4%) se implementaron. Cubrieron productos para tratar 14 enfermedades diferentes. Sin embargo, 137 (77.8%) implicaron medicamentos para la infección del virus de inmunodeficiencia humana y el síndrome de inmunodeficiencia adquirida o enfermedades relacionadas. Resultó que el uso de las flexibilidades de los ADPIC fue más frecuente de lo que comúnmente se espera. Dados los problemas que enfrentan hoy los países en la adquisición de medicamentos de alto precio y patentados, el camino práctico y legal que ofrecen las flexibilidades de los ADPIC para acceder a equivalentes genéricos de costo inferior es cada vez más importante.

## الملخص

يفتقر ملايين من الناس إلى إمكانية الحصول على المستحضرات الصيدلانية الفعالة وخاصة في الدول منخفضة ومتوسطة الدخل، وغالبًا ما يكون ذلك بسبب ارتفاع تكاليف تلك المستحضرات. وقد اعتمد المؤتمر الوزاري لمنظمة التجارة العالمية عام 2001 إعلان الدوحة الخاص بالاتفاقية المتعلقة بجوانب حقوق الملكية الفكرية المتصلة بالتجارة (اتفاق تريبس) والصحة العامة. وكان الإعلان الصادر قد أقر بالآثار المترتبة على حقوق الملكية الفكرية بالنسبة إلى تطوير أدوية جديدة وأسعار الأدوية على حدٍ سواء. كما حدد الإعلان التدابير المعروفة بأوجه مرونة اتفاق تريبس، والتي يستطيع الأعضاء في منظمة التجارة العالمية الاستفادة منها لضمان إمكانية وصول الأدوية للجميع. وتشمل تلك التدابير الترخيص الإلزامي لبراءات اختراع الأدوية وتدابير انتقال المستحضرات الصيدلانية في الدول الأقل نموًا. وكان الهدف من تلك الدراسة يتمثل في توثيق الانتفاع بأوجه مرونة اتفاق تريبس لتوفير الأدوية المكافئة الأقل سعرًا بين عامي 2001 و2016. وبوجه عام، تم الكشف عن 176 من حالات الاستفادة الممكنة لأوجه المرونة في اتفاق تريبس من قبل 89 بلدًا؛ حيث اشتملت 100 حالة منها (بواقع 56.8‏%) على التراخيص الإلزامية أو تراخيص الاستخدام غير التجاري العام، فما تم تسجيل 40 حالة (بواقع 22.7‏%) تم فيها اتباع التدابير الخاصة بانتقال المستحضرات الصيدلانية في الدول الأقل نموًا. وكان المتبقي من الحالات عبارة عن حالة واحدة من الاستيراد الموازي؛ و3 استثناءات بحثية؛ و32 براءة اختراع غير متعلقة بالتدابير. ومن بين الحالات البالغ عددها 176 حالة، تم تطبيق 152 حالة (بواقع ‏‏86.4%). وقد تضمنت منتجات لعلاج 14 مرضًا مختلفًا. وعلى الرغم من ذلك، فقد وردت 137 حالة (أي بنسبة ‏77.8‏%) والتي تعلقت باستخدام الأدوية المخصصة لعدوى فيروس عوز المناعة البشري ومتلازمة نقص المناعة المكتسبة أو الأمراض ذات الصلة. وقد تبين أن الانتفاع بأوجه مرونة اتفاق تريبس أكثر شيوعًا مما كان مفترضًا بشكل عام. وبالنظر إلى المشكلات التي تواجهها الدول اليوم في شراء الأدوية عالية الثمن والحاصلة على براءة اختراع، فإن المسار القانوني والعملي المتاح من خلال جوانب المرونة في اتفاق تريبس لتيسير سبل الحصول على الأدوية المقابلة والمكافئة منخفضة الثمن هو مسار متزايد الأهمية.

## 摘要

数百万人口，尤其是中低收入国家的人们，往往由于负担不起而无法获得足够有效的药品。世界贸易组织 (WTO) 2001 年部长级会议通过了关于《与贸易相关的知识产权协议》(TRIPS) 和公共卫生的《多哈宣言》。该宣言审视了知识产权对新药开发和药品价格的影响。该宣言概述了 WTO 成员为确保所有人都可获得药品而采取的措施，即 TRIPS 灵活性措施。这些措施包括药品专利强制许可和最不发达国家药品流转措施。本研究的目的是记录 2001 年至 2016 年间使用 TRIPS 灵活性措施获取价格较低的非专利药品的情况。总计确认了 89 个国家 176 个可能使用 TRIPS 灵活性措施的案例：100 (56.8%) 例涉及强制许可或公共非商业使用许可证，40 (22.7%) 例援引了最不发达国家的医药流转措施。其余案例为：1 例平行进口；3 例研究异常；和 32 例非专利相关措施。在 176 个案例中，有 152 (86.4%) 例得以执行。它们涵盖了治疗 14 种不同疾病的药品。但是，有 137 (77.8%) 例涉及用于人类免疫缺陷病毒感染和获得性免疫缺陷综合征或相关疾病的药品。我们发现 TRIPS 灵活性措施的使用比通常假定的更为频繁。鉴于当今各国在获取高价专利药品方面所面临的问题，TRIPS 灵活性措施中提供的获取低成本通用药品药物的实用性合法途径变得越来越重要。

## Резюме

Миллионы людей, особенно в странах с низким и средним уровнем дохода, не имеют доступа к эффективным лекарственным средствам, чаще всего по причине их высокой стоимости. На министерской конференции Всемирной торговой организации (ВТО) в 2001 году была принята Дохинская декларация «Соглашение по ТРИПС (Соглашение по торговым аспектам прав на интеллектуальную собственность) и общественное здравоохранение». Декларация подтвердила значение прав на интеллектуальную собственность как для разработки новых лекарственных средств, так и для цен на лекарства. В декларации были изложены меры, известные как гибкие положения ТРИПС, которые могут принять члены ВТО для обеспечения широкого доступа к лекарственным средствам. Эти меры включают обязательное лицензирование патентов на лекарственные средства и переходный период в сфере обращения лекарственных средств в наименее развитых странах. Цель этого исследования заключалась в том, чтобы обосновать использование гибких положений ТРИПС для доступа к недорогим непатентованным лекарственным средствам в период с 2001 по 2016 год. В целом было выявлено 176 случаев возможного использования гибких положений ТРИПС в 89 странах: 100 (56,8%) из них были связаны с обязательным лицензированием или лицензиями на общественное некоммерческое использование, а 40 (22,7%) — с переходным периодом в сфере обращения лекарственных средств в наименее развитых странах. Оставшимися были: 1 случай параллельного импорта, 3 исключения из исследований и 32 случая, не связанные с патентами. Из 176 случаев было реализовано 152 (86,4%). Они охватывали лекарственные средства, предназначенные для лечения 14 различных заболеваний. Однако 137 случаев (77,8%) были связаны с лекарственными средствами, применяемыми при ВИЧ-инфекции (вирус иммунодефицита человека) и синдроме приобретенного иммунодефицита или связанных с ним заболеваний. Было установлено, что гибкие положения ТРИПС применяются чаще, чем это принято считать. С учетом актуальных проблем, с которыми сталкиваются страны при закупке дорогостоящих запатентованных лекарственных средств, все большее значение приобретает практический легальный путь, обеспечиваемый гибкими положениями ТРИПС для доступа к недорогим эквивалентам.

## Introduction

The challenges posed by the high price of antiretroviral medicines in the late 1990s, coupled with widespread patenting of these medicines, led to efforts to ensure that the World Trade Organization’s (WTO’s) Agreement on Trade-Related Aspects of Intellectual Property Rights (TRIPS) could be implemented more flexibly to allow for the procurement of low-priced medicines.^1^ In 2001, a Ministerial Conference of the WTO adopted the Doha Declaration on the TRIPS Agreement and Public Health (that is, the Doha Declaration).^2^ The declaration recognized both the importance of intellectual property for the development of new medicines and concerns that intellectual property rights affected medicine pricing. It lists several measures that countries can take to ensure access to medicines for all, such as the use of compulsory licensing to produce or purchase lower-priced generic medicines. Paragraph 7 of the declaration removed the obligation to grant and enforce medicine patents and data protection for WTO Members designated by the United Nations as least-developed countries, initially until 1 January 2016, this is referred to as the least-developed countries pharmaceutical transition measure. In 2002, the WTO’s Council for TRIPS formally adopted a decision implementing Paragraph 7 and later extended the transition period until at least 2033.^3^^,^^4^ Of the 48 countries designated least-developed countries, 36 are currently WTO Members.^5^

Compulsory licensing is the right granted by a government authority to make use of a patent during the patent term without the consent of the patent holder, for example, for the production or supply of generic medicines. According to Article 31 of TRIPS, a government can also authorize use of a patent for its own purposes: this is called public noncommercial use and is also referred to as government use. A public noncommercial use licence can be assigned either to a state agency or department or to a private entity. When a compulsory licence or public noncommercial use licence is issued, the patent holder is generally entitled to adequate remuneration for use of the patent.^6^

The extent to which countries have deployed TRIPS flexibilities, such as compulsory licences or public noncommercial use licences, for procuring medicines remains underreported. Previous studies have documented well-known and widely publicized cases of compulsory licensing, but have not examined the use of TRIPS flexibilities in procurement.^7^^,^^8^ Moreover, several reports in the literature perpetuate the belief that, since 2001, the use of TRIPS flexibilities has been sporadic and limited.^9^^–^^11^

The aim of our study was to document the use of TRIPS flexibilities to gain access to lower-priced generic medicines. Although we recognized that the TRIPS Agreement offers a range of flexibilities relevant to national pharmaceutical and patenting policies, including the right of countries to define and apply patentability criteria and to refuse to grant patents for certain subject matter (e.g. plants and animals), we focused on measures that can be directly applied to the procurement and supply of medicines. The most relevant measures for increasing access to medicines were: (i) compulsory licensing (including public noncommercial use licensing); (ii) the least-developed countries pharmaceutical transition measure; (iii) parallel importation; and (iv) the research exception. Parallel importation is the importation and resale of a product from another country (where the same product is legitimately on sale at a lower price) without the consent of the patent holder. The research exception refers the use of a patented product or process for research or experimentation without the consent of the patent holder.

## Identifying TRIPS flexibilities

Since 2007, we have been identifying, and collecting information on, instances of the possible use of TRIPS flexibilities internationally and have compiled a database covering the period 2001 to 2016. An instance refers to one of the following events: (i) a government announcement of the intent to invoke a TRIPS flexibility; (ii) a request or application by a third party to invoke a TRIPS flexibility; (iii) the actual use of a TRIPS flexibility; and (iv) a government’s declaration that there are no relevant patents in its territory.

For 164 of the 176 instances we identified, information was available from primary sources, including: (i) patent letters held by procurement agencies, which were not public documents; (ii) legal documents such as licences; and (iii) legal notifications, such as declarations of intent to invoke the least-developed countries pharmaceutical transition measure. These documents were obtained from governments, procurement agencies, law courts and the WTO (that is, as country notifications). Eight other instances were found in the secondary literature^12^^,^^13^ and in official reports,^14^^,^^15^ two instances were identified through personal communications with representatives of nongovernmental organizations who were directly involved in the use of TRIPS flexibilities and could confirm their use (Yunqiong Hu, Médecins sans Frontières, personal communication, 13 October 2014) and one was reported by a civil society organization.^8^ Of the 13 instances for which primary sources were not available, nine involved situations in which the measure was not implemented, which explains the absence of formal legal and government documentation. We verified that we had identified all instances of possible TRIPS flexibility use by searching the LexisNexis, Medline® and Web of Science databases using the search string “compulsory license pharmaceutical” OR “compulsory licence pharmaceutical” OR “compulsory licensing pharmaceutical” OR “government use pharmaceutical” OR “noncommercial use pharmaceutical” and by screening specialized list servers.^16^ This final search yielded one more instance for the database.

We categorized instances of TRIPS flexibility use according to the disease for which the flexibility was invoked and according to the following country classification: (i) developed country; (ii) developing country; (iii) least-developed country; (iv) observer country (that is, a country in WTO accession negotiations); and (v) not a WTO Member. For each instance, we identified the relevant products and verified their patent status using the MedsPaL database (Medicines Patent Pool, Geneva, Switzerland), government documentation and other information in the public domain. This enabled us to determine whether use of a TRIPS flexibility was indeed required to gain access to the generic products; for example, if no valid patent existed, the use of a TRIPS flexibility would not have been necessary. For instances in which the use of a TRIPS flexibility was announced but was not actually used, we collected and analysed information on the reasons for the failure to use it.

## Use of TRIPS flexibility

We collected information on 176 instances of the possible use of TRIPS flexibilities by 89 countries between 2011 and 2016 that were associated with government actions to ensure access to patented medicines (Table 1). Of these, 144 (81.8%) made use of TRIPS flexibility measures: of which 100 involved compulsory or public noncommercial use licences, 40 invoked the least-developed countries pharmaceutical transition measure, 1 involved parallel importation and 3 involved research exceptions. Of the 100 instances of compulsory licensing, 81 were implemented, but 19 were not because: (i) the patent holder offered a price reduction or donation (6 instances); (ii) the patent holder agreed to a voluntary licence allowing the purchase of a generic medicine (5 instances); (iii) no relevant patent existed that warranted the pursuit of the measure (1 instance); (iv) the application was rejected on legal or procedural grounds (5 instances); (v) the applicant withdrew the application (1 instance); and (vi) the application has been pending since 2005 with no response (1 instance). The least-developed countries pharmaceutical transition measure was invoked in 40 instances by a total of 28 countries. However, 2 of the 28 countries were developing countries that invoked the measure erroneously, 3 were observer countries and 1 was not a WTO Member. The 3 research exceptions involved generic medicines used in clinical studies. In the remaining 32 instances, governments used measures not related to patents (Table 1). In 26 of the 32, countries informed the supplier that there was no relevant patent in their territory. However, this was only the case in 4 of the 26. The other 6 instances involved import authorizations for products that did not refer to the patent status of the products: 4 concerned the importation of a product for which patents existed in the territory and 2 concerned countries that were not WTO Members. Overall, TRIPS flexibilities were implemented in 152 of the 176 instances identified (86.4%).

**Table 1:** Measures used by governments to gain access to lower-priced generic medicines, 2001–2016

Type of measure	Instances of use, no. (%)
**TRIPS flexibility**	
Compulsory licence	48 (27.3)
Public noncommercial use (government use) licence	52 (29.5)
Least-developed countries pharmaceutical transition measure	40 (22.7)
Parallel importation	1 (0.6)
Research exception	3 (1.7)
**Non-patent-related measure**	
Declaration of no patent in territory	26 (14.8)
Import authorization without reference to patent status	6 (3.4)
**Total**	**176 (100.0)**

TRIPS: Trade-Related Aspects of Intellectual Property Rights (Agreement on).

**Fig. 1:**
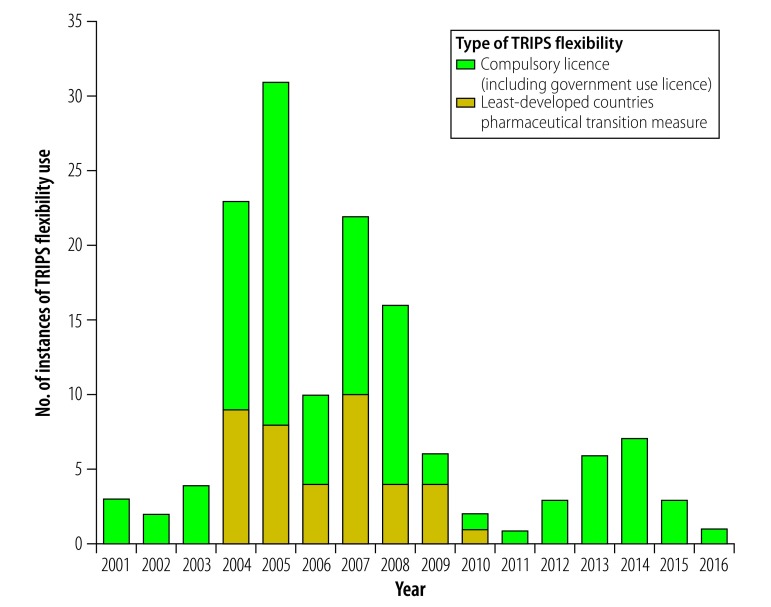
Use of Trade-Related Aspects of Intellectual Property Rights flexibilities to gain access to lower-priced generic medicines, worldwide, 2001–2016

The 176 instances covered products for treating 14 different diseases. Table 2 summarizes how often TRIPS flexibilities were used for different diseases according to the country’s WTO classification. Of the 140 instances in which either compulsory licences, public noncommercial use licences or the least-developed countries pharmaceutical transition measure was used, 103 (73.6%) concerned human immunodeficiency virus (HIV) infection and acquired immune deficiency syndrome (AIDS) or related diseases. For 25% (10/40) of instances in which the least-developed countries pharmaceutical transition measure was used, the flexibility was invoked for all medicines. A TRIPS flexibility was used for cancer medications in 6.8% (12/176). Fig. 1 shows the variation in the number of instances of TRIPS flexibility use over time: use of compulsory licences, public noncommercial use licences and the least-developed countries pharmaceutical transition measure peaked between 2004 and 2008.

**Table 2:** Measures used by governments to gain access to lower-priced generic medicines, by country classification and disease, worldwide, 2001–2016

Country classification and disease	Measure used to gain access to lower-priced generic medicines, no. (%)						
	TRIPS flexibility				Non-patent-related measure		Total
	Compulsory licence^a^	Least-developed countries pharmaceutical transition measure^b^	Parallel importation	Research exception^c^	Declaration of no patent in territory	Import authorization^d^	
**Developed countries**							
HIV^e^	2 (1.1)	0 (0.0)	0 (0.0)	0 (0.0)	0 (0.0)	0 (0.0)	2 (1.1)
Cancer	1 (0.6)	0 (0.0)	0 (0.0)	0 (0.0)	0 (0.0)	0 (0.0)	1 (0.6)
Other	6 (3.4)	0 (0.0)	0 (0.0)	0 (0.0)	0 (0.0)	0 (0.0)	6 (3.4)
**Developing countries**							
HIV^e^	51 (29.0)	1 (0.6)	0 (0.0)	3 (1.7)	17 (9.7)	4 (2.3)	76 (43.2)
Cancer	11 (6.3)	0 (0.0)	0 (0.0)	0 (0.0)	0 (0.0)	0 (0.0)	11 (6.3)
Other	9 (5.1)	1 (0.6)	1 (0.6)	0 (0.0)	1 (0.6)	0 (0.0)	12 (6.8)
**Least-developed countries^f^**							
HIV^e^	12 (6.8)	26 (14.8)	0 (0.0)	0 (0.0)	0 (0.0)	0 (0.0)	38 (21.6)
Cancer	0 (0.0)	0 (0.0)	0 (0.0)	0 (0.0)	0 (0.0)	0 (0.0)	0 (0.0)
Other	0 (0)	8 (4.5)	0 (0.0)	0 (0.0)	0 (0.0)	0 (0.0)	8 (4.5)
**WTO observer countries^g^**							
HIV^e^	7 (4.0)	2 (1.1)	0 (0.0)	0 (0.0)	6 (3.4)	2 (1.1)	17 (9.7)
Cancer	0 (0.)	0 (0.0)	0 (0.0)	0 (0.0)	0 (0.0)	0 (0.0)	0 (0.0)
Other	0 (0.0)	1 (0.6)	0 (0.0)	0 (0.0)	0 (0.0)	0 (0.0)	1 (0.6)
**Not WTO Members**							
HIV^e^	1 (0.6)	1 (0.6)	0 (0.0)	0 (0.0)	2 (1.1)	0 (0.0)	4 (2.3)
Cancer	0 (0.0)	0 (0.0)	0 (0.0)	0 (0.0)	0 (0.0)	0 (0.0)	0 (0.0)
Other	0 (0.0)	0 (0.0)	0 (0.0)	0 (0.0)	0 (0.0)	0 (0.0)	0 (0.0)
**Total**	**100 (56.8)**	**40 (22.7)**	**1 (0.6)**	**3 (1.7)**	**26 (14.8)**	**6 (3.4)**	**176 (100.0)**

HIV: human immunodeficiency virus; TRIPS: Trade-Related Aspects of Intellectual Property Rights (Agreement on); WTO: World Trade Organization.

^a^ Compulsory licences included public noncommercial use (or government use) licences issued in accordance with Article 31 of the Agreement on Trade-Related Aspects of Intellectual Property Rights (TRIPS).

^b^ Paragraph 7 of the Doha Declaration removed, for a transitional period, the obligation to grant and enforce medicines patents for World Trade Organization (WTO) Member States designated by the United Nations as least-developed countries.

^c^ In accordance with Article 30 of TRIPS.

^d^ Import authorization without reference to patent status.

^e^ HIV, acquired immune deficiency syndrome and related diseases.

^f^ WTO Member States designated least-developed countries by the United Nations.

^g^ Countries in negotiations for accession to the WTO.

Note: Countries were categorized as developed or developing according to WTO classification.

TRIPS: Trade-Related Aspects of Intellectual Property Rights (Agreement on).

Note: The least-developed countries pharmaceutical transition measure applies to World Trade Organization (WTO) Member States designated by the United Nations as least-developed countries and removes them from the obligation to grant and enforce medicine patents in accordance with Paragraph 7 of the Doha Declaration.

## Discussion

Our study found that countries made extensive use of TRIPS flexibilities between 2001 and 2016. This was previously unreported. The most frequently used measures were compulsory licensing, public noncommercial use licensing and the least-developed countries pharmaceutical transition measure, which together accounted for 79.5% (140/176) of instances. To date, the most comprehensive, published database lists 34 potential compulsory licences in 26 countries.^9^ We also documented 26 instances in which generic medicines were procured after a declaration that there was no relevant patent in the territory. Strictly, this is not a TRIPS flexibility. However, generic medicines were procured despite patents actually being registered in 22 of the 26 instances. All concerned HIV medications, which points to a more flexible attitude towards the protection of intellectual property in the context of the global response to the HIV epidemic. In the majority of instances we identified, the application of a TRIPS flexibility was driven by the procurement of medicines for the treatment of HIV/AIDS and related diseases.

In 1997, the World Health Organization (WHO) published the first guide for Member States on how to comply with TRIPS while limiting the negative effect of patent protection on medicine availability.^17^ The political momentum of WHO’s “3 by 5” initiative for HIV treatment combined with HIV treatment campaigns and new funding from governments, the Global Fund to Fight AIDS, Tuberculosis and Malaria and the United States’ President's Emergency Plan for AIDS Relief enabled countries to scale up the procurement of antiretroviral medicines. In addition, new global funding mechanisms incorporated procurement guidelines that encouraged countries to purchase low-priced medicines. The Global Fund, for example, urged its recipients “to attain and to use the lowest price of products through competitive purchasing from qualified manufacturers.” The Global Fund also specifically encouraged “recipients in countries that are WTO Members to use the provisions of the TRIPS Agreement and interpreted in the Doha Declaration, including the flexibilities therein, to ensure the lowest possible price for products of assured quality.”^18^ Furthermore, World Bank guidelines on the procurement of HIV medicines provided governments with practical advice on how to use various TRIPS flexibilities.^19^

Antiretroviral medicines were the first class of new essential medicines that were widely patented and, when the drugs were introduced, medicines procurement agencies did not have experience with the supply of such products. In the late 1990s, concerns about possible patent infringement lawsuits were common among medicine suppliers. In fact, several legal disputes broke out.^20^ Procurement agencies in sub-Saharan Africa that supplied generic HIV medicines were threatened with legal action by patent holders.^21^^,^^22^ Consequently, these agencies sought assurances that they could supply antiretrovirals without the risk of legal action. The Doha Declaration offered much-needed clarification on the legal rights of WTO Members with regard to intellectual property and public health and, subsequently, provided an important basis for these assurances. The declaration was also a vital political statement of support for countries that were struggling to provide access to expensive medicines while complying with the TRIPS Agreement.^23^

Increased funding for HIV treatment largely explains the rise in the number of instances of TRIPS flexibility use after 2003 (Fig. 1). In fact, use of these flexibilities helped create and sustain the generic competition that brought down the price of HIV medicines. By 2008, 95% (by volume) of the global donor-funded antiretroviral market comprised generic medicines, which primarily came from India, where these medicines were not patented. Moreover, Indian generic manufacturers produced fixed-dose combinations of antiretrovirals that were not available elsewhere.^24^ Some companies had issued non-assert declarations (that is, commitments not to enforce their patents) by 2008^25^ or engaged in voluntary licensing, often in response to the threat of a compulsory licence. After 2008, the use of TRIPS flexibilities for HIV/AIDS treatment decreased because voluntary licensing had become more common. In 2010, the Medicines Patent Pool was founded with the support of Unitaid. The patent pool negotiated voluntary licences that enabled the production and supply of generic HIV medicines and, as a result, countries within the territorial scope of Medicines Patent Pool licences no longer needed to invoke TRIPS flexibilities for HIV treatments. By the end of 2017, the territorial scope of these licences covered 87% to 91% of adults and 99% of children living with an HIV infection in developing countries.^26^ In 2016, 93% of people with an HIV infection who had access to antiretrovirals used generic products.^27^ This would not have been the case if the decreased use of TRIPS flexibilities led to countries switching back to the originators’ branded products. Today, the scope of the Medicines Patent Pool also covers hepatitis C virus infection and tuberculosis. The *Lancet* Commission on Essential Medicines Policies recommended that all new essential medicines should be covered by the work of the Medicines Patent Pool.^28^

Interestingly, most instances of TRIPS flexibility use documented in our study were invoked and implemented as part of day-to-day procurement and took place without much publicity. This was very effective, especially for the supply of new generic HIV medicines. The relatively unknown use of TRIPS flexibilities for regular drug procurement that we uncovered is in stark contrast to the publicity attracted by some instances of their use by middle-income countries. For example, the compulsory licences issued by Brazil, India and Thailand caused a widespread controversy because of the harsh responses they provoked by the United States of America and the European Union,^29^^–^^31^ both of which discouraged the uptake of TRIPS flexibilities.^32^ In 2012, a compulsory licence issued by India for a cancer medicine provoked an out-of-cycle review by the Office of the United States Trade Representative.^33^ In 2016, Colombia sought support from WHO to issue a compulsory licence for the cancer drug imatinib, which is included in WHO’s model list of essential medicines.^34^ The country came under strong pressure from Switzerland and the United States to abandon its plans for the licence, with United States’ officials threatening to withdraw financial support for Colombia’s peace process.^35^ These disputes show that effective use of TRIPS flexibilities remains politically sensitive. However, an important observation of ours is that the majority of TRIPS flexibilities invoked were actually successfully implemented.

Lessons can be learnt from antiretroviral procurement practices for other, patented and highly priced, new, essential medicines. The globalization of intellectual property norms through international trade law means that new essential medicines for diseases such as cancer, tuberculosis and hepatitis C virus infection will probably be widely patented.^28^ In 2015, for example, WHO added several new, high-priced medicines to its model list of essential medicines. Initiatives by pharmaceutical companies to increase access to medicines outside the field of HIV/AIDS are weak and predominantly based on donations or small-scale, patient-based price discounts.^36^ In addition, global funding is lacking for medicines for diseases other than HIV infection, tuberculosis and malaria, which increases the importance of efficient access to lower-cost medicines. Furthermore, with increasingly widespread pharmaceutical patenting, the use of TRIPS flexibilities is becoming more relevant and urgent.

Our study shows that governments have successfully used public noncommercial use licences and the least-developed countries pharmaceutical transition measure to procure patented medicines, thereby providing suppliers of generic products with the required legal assurances. The use of standard licence models would streamline the process of procuring generic medicine equivalents of new expensive patented medicines.^37^

The use of TRIPS flexibilities is also important for countries excluded from voluntary licences, including Medicines Patent Pool licences. For example, generic medicines produced under certain voluntary licences may be supplied to a country outside the scope of that licence if that country has issued a compulsory licence.^38^ In addition, TRIPS flexibilities remain important for diseases for which voluntary licences or other access initiatives do not exist at present, such as cancer and other noncommunicable diseases.

Government noncommercial use of patents in medicines procurement is not new. In the 1960s and 1970s, some European governments and the United States routinely used this method. Today, calls by high-income countries to reinstate this measure to battle high medicine prices are getting louder, for example, in Chile, France, Ireland, the Netherlands, the United Kingdom of Great Britain and Northern Ireland and the United States.^39^^–^^44^ In 2017, with only a veiled reference to the high price of hepatitis C virus medicines, the Italian government gave its citizens the right to import more-affordable generic versions for their personal use.^45^ In 2016, the German Federal Patent Court issued a compulsory licence for the antiretroviral medicine raltegravir, citing the urgent public interest of patients and health risks associated with the potential nonavailability of the drug.^46^

The use of TRIPS flexibilities is an important tool that can help countries fulfil their human rights obligation to provide access to essential medicines as part of the progressive realization of the right to health.^47^ Alongside the legal obligations of states, pharmaceutical companies also have a responsibility to provide access to medicines, for example, through voluntary licensing.^48^ The Medicines Patent Pool could be expanded to include all new essential medicines so that these medicines would be available as generics in low- and middle-income countries well before the patents expire. In the absence of voluntary or Medicines Patent Pool licences, governments could use TRIPS flexibilities as part of regular procurement.

Regrettably, although the need for government resolve and action to bring down the price of patented medicines is growing, the policy space to do so is narrowing because of TRIPS-plus provisions included in trade agreements.^49^ These TRIPS-plus provisions render the flexibilities in the TRIPS Agreement, such as compulsory licensing, less effective by placing restrictions on their use. One example is that the grounds for compulsory licensing could be limited to emergencies, which would make their use in regular procurement nearly impossible. Further, political responses in high-income countries to the use of TRIPS flexibilities by certain middle-income countries has been a substantial obstacle to their routine use.^35^ A strong political response to the plans of only a few countries to issue compulsory licences for cancer medications is likely to have a chilling effect on others.

In conclusion, our study shows that TRIPS flexibilities have been used more frequently than is commonly assumed and have proven effective for procuring generic versions of essential medicines, particularly for treating HIV infection. Given the problems many countries face today in providing access to high-priced, patented medicines, TRIPS flexibilities are increasingly important. However, their use should not be regarded as a measure of last resort because they can be considered for the routine procurement of generic versions of expensive, new, essential medicines, while providing adequate remuneration to the patent holder. Their use will help create and sustain the generic competition that has been effective in bringing down the price of medicines and that could help ensure universal access to new, essential medicines for all.
